# Effect of dual-task interference on the hand flexibility of patients with Parkinson’s disease carrying the leucine-rich repeat kinase 2 gene mutation

**DOI:** 10.3892/etm.2013.1352

**Published:** 2013-10-18

**Authors:** GUANGAN ZHOU, SHEN YANG, TUANZHI CHEN, PIU CHAN, YIFENG DU

**Affiliations:** 1 Department of Neurology, Provincial Hospital Affiliated to Shandong University, Jinan, Shandong 250021, P.R. China; 2 Department of Neurology, Taian City Central Hospital, Taian, Shandong 271000, P.R. China; 3 Department of Neurology, Liaocheng People’s Hospital, Liaocheng Clinical School of Taishan Medical University, Liaocheng, Shandong 252000, P.R. China; 4 Department of Neurology, Xuanwu Hospital Attached to the Capital Medical University, Beijing 100053, P.R. China

**Keywords:** Parkinson’s disease, leucine-rich repeat kinase 2 gene, Purdue pegboard, dual-task interference

## Abstract

The aim of the present study was to observe the changes in hand flexibility of patients with Parkinson’s disease (PD) and the effect of dual-task interference. Patients with PD were distributed into two subgroups: the leucine-rich repeat kinase 2 (LRRK2) mutation PD (LRRK2^+^) and the LRRK2 mutation-free (LRRK2^−^) PD groups. The healthy controls were distributed into two subgroups: the LRRK2^+^ control and the LRRK2^−^ control groups. The first task was the Purdue pegboard test. The second task was to perform serial seven subtractions. Single-task and dual-task tests were performed, respectively. The numbers of pegs inserted with the dominant hand, non-dominant hand and both hands in the pegboard test and the number of correct responses in the serial seven subtractions test within 30 sec were recorded. The United Parkinson’s Disease Rating Scale (UPDRS) III score of examinees in the LRRK2^+^ PD group was significantly higher than that of examinees in the LRRK2^−^ PD group (P<0.05). The number of pegs inserted within 30 sec by patients with PD was significantly lower than that by the controls (P<0.05). The indicators of patients with PD, including number of variation in the subtraction test score when the dominant-hand was used in the pegboard test (NVD), number of variation in the subtraction test score when the non-dominant hand was used in the pegboard test (NVND) and number of variation in the subtraction test score when the both-hand was used in the pegboard test (NVB), were significantly different compared with those of the control group (P<0.05). The difference in the number of correct responses within 30 sec of patients with PD was significantly correlated with the UPDRSIII score (P<0.05). In conclusion, the hand flexibility of patients with PD was markedly lower than that of the controls. When both tasks were performed, the ability markedly decreased in the second cognitive task, particularly in the LRRK2^+^ PD group.

## Introduction

Parkinson’s disease (PD) is a type of chronic progressive degenerative disease of the nervous system. According to the diagnostic criteria of the UK Brain Bank (1), PD mainly manifests as motor symptoms, including resting tremor, tonic bradykinesia or postural instability (2).

The hand flexibility of patients with PD decreases, which seriously affects daily activities, including the ability to use a keyboard, buttoning, writing and tying shoes. Numerous quantitative motor examinations have shown that patients with PD have various hand motor dysfunctions, including repetitive movement of a single finger (3), coordinated movement of fingers (4), repetitive movement of the whole hand (5), coordinated movement of the hand and wrist (6) and coordinated movement of the hand and forearm as well as the upper arm (7).

During daily life, dual tasks or multiple tasks are encountered simultaneously, such as walking while chatting and writing while listening to music. A dual-task model is able to disperse the examinees’ attention through the first task and thus produce a more natural environment. Behavioral competence in this environment is more representative than a single task. The dual-task model is widely used to examine the effect of distraction on executive function (8). When patients with PD simultaneously perform movement and cognitive tasks, movement tasks that depend on guidance by the visual sense are not damaged markedly. Cognitive tasks that do not depend on guidance by the visual sense are damaged markedly (9). Among various inspection devices of hand movement function, the Purdue pegboard is universally used for the evaluation of hand movement function, due to its simplicity, short inspection time and relatively good sensitivity and specificity (10).

Numerous susceptibility genes of PD have been identified. Among them, the leucine-rich repeat kinase 2 (LRRK2) gene is the most common risk gene of sporadic PD and familial PD. In 2008, Healy *et al*(8) studied patients with PD from 21 different districts and observed that the LRRK2 G2019S mutation was common in Jewish individuals in Northern Europe and North Africa, but rare in Asian individuals. In mainland China, Taiwan, Hong Kong and Japan, the G2385R mutation accounts for 10% of patients with PD and the onset risk of PD increases two-fold (11). G2385R and R1628P mutations of LRRK2 in patients with PD in China are relatively common. Through the study of LRRK2 gene polymorphism in patients with PD in mainland China, Yu *et al*(12) observed that the incidence of the R1628P site was 5.2% and the onset risk of PD increased 2.678-fold. Currently, there are relatively few studies concerning patients with PD who carry the LRRK2G2385R and R1628P gene mutations. To the best of our knowledge, the effect of dual-task interference on the hand flexibility of LRRK2^−^ PD patients has not been reported.

In this study, the hand flexibility changes of LRRK2^+^ and LRRK2^−^ PD patients who carried G2385R and R1628P gene mutations were studied using single and dual tasks in order to investigate their differences.

## Materials and methods

### Research subjects

The total number of subjects was 122. In the early stage of longitudinal study of Parkinson’s disease in China, LSPDC, these patients accepted genetic screening for G2385R and R1628P of the risk gene LRRK2. There were 46 cases of PD obtained from the ‘National 863’ database. Among them, there were 22 cases of PD who carried the LRRK2 gene mutation (G2385R and G1628P) and there were 24 cases of PD who did not carry the LRRK2 gene mutation. There were 76 healthy controls. Healthy individuals who matched the age and gender of the PD patients were recruited from the community elderly database in Beijing. Among them, there were 38 healthy subjects carrying the LRRK2 gene mutation and 38 healthy subjects who did not carry the LRRK2 gene mutation. The PD patients were diagnosed by PD experts. PD diagnosis was in accordance with the diagnostic criteria for PD of the Parkinson’s UK Brain Bank. The inclusion criteria were as follows: all subjects accepted Mini-Mental Status Examination (MMSE; score 0–30) and the evaluation was within the normal range. Patients were without other nervous system diseases. The patients included in the study signed an informed consent form.

The medical ethics certificate was approved by the Medical Ethics Committee of Xuanwu Hospital of Capital Medical University (Beijing, China).

### Grouping

According to whether patients with PD carried the LRRK2 gene mutation, they were divided into two subgroups: LRRK2^+^ PD (patients with PD who carried the LRRK2 mutation) and LRRK2^−^ PD (patients with PD who did not carry the LRRK2 mutation). According to whether healthy controls carried the LRRK2 mutation, the controls were divided into two subgroups: the LRRK2^+^ control (healthy subjects who carried the LRRK2 mutation) and the LRRK2^−^ control groups (healthy subjects who did not carry the LRRK2 mutation).

### Research methods

The included PD patients accepted standardized neurological examination and clinical assessment. Demographic information and PD history information were collected face-to-face. The motor function of patients was assessed using the United Parkinson’s Disease Rating Scale (UPDRS) III. The progress of PD was evaluated using the Hoehn and Yahr stage scale (H-Y) (13). The cognitive function of the patients was evaluated by MMSE. The depressive state of the patients was evaluated using the Hamilton Depression Scale-17 (HAMD-17).

The first task was the Purdue pegboard test, which mainly reflected the speed and flexibility of hand movement. Purdue pegboard 32020, produced by Lafayette Instrument Company (Lafayette, IN, USA), was used for the test. The test was performed according to the standardized method. The subjects were asked to insert the pegs from ipsilateral small bowls into holes on the board one by one with the right hand, left hand and both hands within 30 sec, respectively. The test was performed three times and the mean was used. The dominant hand score (PPTD), non-dominant hand score (PPTND) and both hands score (PPTB) were obtained.

The second task was serial seven subtractions. The initial number was randomly selected from a set of data cards marked with the numbers 290–310. The card marked with the used number was removed from the rest of the data cards. When the pegboard test was started, the selected initial number was shown simultaneously. Then the subjects counted backwards every 7th number. A voice recorder and timer were used to carry out the recording. Single task seven subtractions, dual-task seven subtractions with the dominant hand, dual-task seven subtractions with the non-dominant hand and dual-task seven subtractions with both hands were performed, respectively. The number of correct responses within 30 sec was recorded (if seven was subtracted from the previous number correctly, it was regarded as a correct calculation). Single task score (NU), dominant hand score (ND), non-dominant hand score (NND) and both hands score (NB) were obtained.

The difference in the performance in the pegboard test between single and dual tasks was calculated according to the formula: Change in peg number in dual tasks = (peg number in single task - peg number in dual tasks)/peg number in single task. Accordingly, a dominant hand score (PVD), non-dominant hand score (PVND) and both hands score (PVB) were calculated.

The difference in the performance in the subtractions test between single and dual tasks was calculated according to the formula: Change in correct response number of dual tasks = (correct response number of single task - correct response number of dual tasks)/correct response number of single task. Accordingly, a dominant hand score (NVD), non-dominant hand score (NVND) and both hands score (NVB) were calculated.

In the single task pegboard test, subjects were asked to insert pegs as quickly as possible within 30 sec. The instruction of the single task calculation test was to perform serial seven subtractions as quickly as possible with 30 sec. The dual-task test instruction was to insert pegs and perform serial seven subtractions as quickly as possible within 30 sec.

### Sequence and timing of tests

The tests were carried out in a laboratory environment. The patients with PD were tested in the best drug reaction period (on stage). The evaluation sequences of all subjects were the same. Firstly, demographic information was registered, and MMSE and UPDRSIII were assessed. Secondly, single task and dual-task tests were performed.

### Statistical analysis

SPSS 18.0 software (SPSS, Inc., Chicago, IL, USA) was used for statistical analysis of the data. One-way ANOVA was used for the comparisons among groups. An independent-sample Student’s t-test was used for comparisons between groups. A Chi-square test was used for comparisons of enumeration data. A paired Student’s t-test was used for comparisons between single task and dual tasks of patients in the same group and the correlation between them was analyzed by Pearson linear correlation. Linear regression analysis was used to control the effect of influencing factors on the first and second task. P<0.05 was considered to indicate a statistically significant difference.

## Results

### Demographic characteristics of the subjects and disease characteristics of patients with PD

As shown in Table I, there were 22 cases of LRRK2^+^ PD, 24 cases of LRRK2^−^ PD, 38 LRRK2^+^ normal controls and 38 LRRK2^−^ normal controls. The compositions of age and gender of the four groups were similar: the age ranged between 45 and 81 years in the LRRK2^+^ PD group, with an average age of 54.37±5.30 years. The age ranged between 49 and 84 years in the LRRK2^−^ PD group, with an average age of 53.50±9.04 years. In the LRRK2^+^ control group, there were 24 males and 14 females, and the age ranged between 45 and 81 years, with an average age of 56.86±7.96 years. In the LRRK2^−^ control group, there were 24 males and 14 females, and the age ranged between 49 and 84 years, with an average age of 56.20±10.48 years. There were 13 males (59.1%) in the LRRK2^+^ PD group, 14 males (58.33%) in the LRRK2^−^ PD group, 24 males (63.16%) in the LRRK2^+^ control group and 14 males (8.33%) in the LRRK2^−^ control group. The differences in age, gender, educational level and MMSE score between the four groups were not statistically significant, but comparable. The average scores of HAMD of patients with PD in the two groups were significantly higher than those of the normal control groups (P<0.001). The differences in the HAMD score and H-Y stage of the two groups of patients with PD were not statistically significant. However, the difference in UPDRSIII score between the two groups was statistically significant (P<0.05). The UPDRSIII score of the LRRK2^+^ PD group was markedly higher than that of the LRRK2^−^ PD group.

### Numbers of pegs inserted within 30 sec by different subjects during single and dual tasks

The differences in the number of pegs inserted within 30 sec among the four groups, as shown in Table II, were compared by variance analysis. An independent-sample Student’s t-test was used to compare the differences between groups. Linear regression analysis was used to control for the effect of HAMD. Whether asked to perform the single pegboard task or to perform two tasks at the same time, the numbers of pegs inserted within 30 sec in the LRRK2^+^ PD and the LRRK2^−^ PD groups were lower than those in the control groups. The single-task PPTD, single-task PPTND, single-task PPTB, dual-task PPTD, dual-task PPTND and dual-task PPTB of the two PD groups were all significantly lower than those in the normal control groups (P<0.05). The difference in the number of pegs inserted within 30 sec between the LRRK2^+^ PD and LRRK2^−^ PD groups, as well as between the LRRK2^+^ control and LRRK2^−^ control groups, was not significant.

A paired Student’s t-test was used to compare the differences of various indicators between single and dual tasks. The numbers of pegs inserted within 30 sec with the dominant hand, non-dominant hand and both hands during dual-task were all lower than those during single-task, but the differences were not significant.

### Number of correct responses within 30 sec of different subjects during single and dual tasks

The differences in the number of correct responses within 30 sec among the four groups, as shown in Table III, were compared by variance analysis. An independent-sample Student’s t-test was used to compare the differences between groups. Linear regression analysis was used to control for the effect of HAMD. When the single serial seven subtractions task was performed, the differences in the number of correct responses within 30 sec between groups were not significant. When dual tasks were performed, the numbers of correct responses within 30 sec in the LRRK2^+^ PD and the LRRK2^−^ PD groups were lower than those in the normal control groups. ND, NND and NB were all significantly lower than those in the normal control groups (P<0.05). The difference in the number of correct responses within 30 sec between the LRRK2^+^ PD and the LRRK2^−^ PD groups, as well as the LRRK2^+^ control and the LRRK2^−^ control groups, was not significant.

A paired Student’s t-test was used to compare the differences of various indicators between single and dual tasks. When dual tasks were performed, various indicators, including ND, NND and NB, in the LRRK2^+^ PD and the LRRK2^−^ PD groups were significantly lower than those for the single task (P<0.05). The differences of indicators in the LRRK2^+^ control and LRRK2^−^ control groups between single task and dual tasks were not significant. Since dual-task seven subtractions with the dominant hand was performed first, the number of correct responses within 30 sec during dual-task seven subtractions with both hands was higher than the baseline value of single task seven subtractions.

### Comparisons of differences in the numbers of inserted pegs and correct responses within 30 sec when a single task and dual tasks were performed

The differences in the number of pegs inserted within 30 sec and the number of correct responses between single and dual tasks performed by different subjects in the four groups, as shown in Table IV, were compared by variance analysis. An independent-sample Student’s t-test was used to compare the differences between groups. Linear regression analysis was used to control for the effect of HAMD. The differences in various indicators of pegboard test performance between single task and dual tasks, i.e., PVD, PVND and PVB, were not significantly different among the groups. The changes in the number of correct responses in the LRRK2^+^ PD and the LRRK2^−^ PD groups were larger than those in the normal control groups. Indicators of changes in performance in the serial seven subtractions test between single and dual tasks, i.e., NVD, NVND and NVB, were significantly different in the PD groups compared with those of the control groups (P<0.05). The change in the number of correct responses in the LRRK2^+^ PD group was larger than that in the LRRK2^−^ PD group and the differences in indicators, i.e., NVD, NVND and NVB, were statistically significant (P<0.05). The differences in the indicators NVD, NVND and NVB between the LRRK2^+^ control and the LRRK2^−^ control groups were not significant.

### Analysis of the correlation between UPDRSIII score and the difference in correct response number within 30 sec between single and dual tasks

The difference in the number of correct responses within 30 sec between single and dual tasks of patients with PD was significantly correlated with UPDRSIII score. The Pearson correlation coefficients in the LRRK2^+^ PD group were 0.687, 0.641 and 0.622 for NVD, NVND and NVB, respectively (P<0.001). The Pearson correlation coefficients in the LRRK2^−^ PD group were 0.663, 0.557 and 0.549, respectively (P<0.05; Table V).

## Discussion

PD is a common geriatric disease. Nigral dopaminergic neuron degeneration and necrosis, and the formation of Lewy bodies are the main manifestations of pathological changes. This leads to a reduction of striatal dopamine content. However, the acetylcholine system function is relatively sthenic. Thus clinical manifestations of PD, including resting tremor, stiffness, bradykinesia and gait abnormalities, occur (14). UPDRSIII is the most commonly used scale in the screening of patients with PD. The pegboard test has a relatively high sensitivity and specificity, and it is simple and practicable. Therefore, hand flexibility of subjects was evaluated with combined application of UPDRSIII and the pegboard test.

The present study demonstrated that there were certain differences in motor symptoms of patients between the LRRK2^+^ PD and the LRRK2^−^ PD groups. The UPDRSIII score of patients in the LRRK2^+^ PD group was significantly higher than that in the LRRK2^−^ PD group. This was generally in accordance with previous studies (15). In 2011, Marras *et al*(16) studied the LRRK2^+^ PD phenotype and observed that tremor and dystonia were more common in the motor symptoms of patients with PD who carried the LRRK2 (G2019S) gene mutation. Cognitive disorder and dysosmia were rarer than sporadic PD. Johansen *et al*(14) studied 47 first-degree healthy relatives of the LRRK2^+^ PD patients and observed that healthy LRRK2 (G2019S, N1437H) mutation carriers had subclinical motor symptoms and non-motor symptoms of PD. In the current study, it was demonstrated that there was no difference in UPDRSIII score between the LRRK2^+^ control and the LRRK2^−^ control groups. The reason for the lack of concordance with the previous studies may be that the mutation sites of LRRK2 in the subjects in this study were G2385R and G1628P, while the mutation sites of LRRK2 in previous studies were different.

The reduction in hand flexibility of patients with PD seriously affects the daily life of patients and aggravates the patient’s physiological and psychological burden. In this study, it was demonstrated that, whether asked to perform a single pegboard task or to perform dual tasks simultaneously, the numbers of inserted pegs within 30 sec in the LRRK2^+^ PD and the LRRK2^−^ PD groups were markedly lower than those in the normal control groups. The hand flexibility of patients with PD decreased. The indicators of changes in pegboard test performance, i.e., PVD, PVND and PVB, were not markedly different between groups. This was in accordance with previous research results (17). Haaxma *et al*(7) performed a series of timed exercise tests in 107 patients with PD. Stride length, writing time and space, results of the pegboard test with one hand and both hands, results of the finger tapping test and the alternate motion of PD patients were significantly different from those of healthy controls. Müller *et al*(18) studied 157 cases of PD in the lowest drug reaction period and observed that the patients’ abilities to perform a single tapping task or pegboard test were markedly decreased. The two tests were considered to be effective indicators for the objective assessment of disease severity. The pegboard test had greater correlation with disease severity.

In the present study, it was observed that the numbers of correct responses within 30 sec in the LRRK2^+^ PD and LRRK2^−^ PD groups were lower than those in the normal control groups. Indicators, including ND, NND and NB, were significantly lower than those in the normal control groups (P<0.05). The difference in the number of correct responses between single and double tasks in the LRRK2^+^ PD and LRRK2^−^ groups were larger than those in the normal control group. That was in accordance with previous research results (9). The causes may be as follows. The capability of attention of PD patients were certain. More attention was given to the first task, which depended on vision. Insufficient attention was given to the second cognitive task. Dual-task interference refers to the phenomenon that when an individual performs two tasks at the same time, the achievement in one or both tasks is damaged (19). The results of dual-task interference are affected by numerous factors, including the nature of the tasks, individual factors, cognitive factors and environmental factors (20,21).

A number of new discoveries were made in the current study. The hand flexibility of PD patients decreased markedly, however, indicators of changes in pegboard performance between single and dual tasks, i.e., PVD, PVND and PVB, in the groups were not significantly different. When dual tasks were performed, the patients’ ability to perform the second cognitive task decreased markedly, particularly in the patients in the LRRK2^+^ PD group. The difference in the number of correct responses within 30 sec between single and dual tasks was markedly correlated with the UPDRSIII score. The reason for these differences may be that the attention ability of patients in the LRRK2^−^ PD group is weaker. However, further study of the cognitive functions of patients with LRRK2^+^ PD and dual-task interference are required.

In conclusion, compared with control groups, the hand flexibility of patients with LRRK2^+^ PD and LRRK2^−^ PD was markedly decreased. When dual tasks were performed, the change in hand flexibility from that in the single task was not markedly different between the LRRK2^+^ PD and the LRRK2^−^ PD groups, while the ability to perform the second cognitive task decreased markedly decreased in the LRRK^+^ PD group. The novel results of this study were as follows: the UPDRSIII score of patients with LRRK2^+^ PD is significantly higher than that of patients with LRRK2^−^ PD. The ability of patients with PD to perform the second cognitive task was markedly decreased, particularly in the patients with LRRK2^+^ PD.

## Figures and Tables

**Table I:** Demographic information and movement characteristics in PD patients and controls.

	Groups			
	
Characteristics	LRRK2^+^ PD	LRRK2^−^ PD	LRRK2^+^ control	LRRK2^−^ control
Age (years)	54.37±5.30	53.50±9.04	56.86±7.96	56.20±10.48
Gender (male/female)	13/9	14/10	24/14	24/14
Educational level (years total)	10.69±2.81	9.83±2.25	12.37±2.51	12.55±3.44
MMSE score	27.62±1.21	27.55±1.40	28.57±0.98	28.53±1.29
HAMD score	7.56±2.39^a^	7.87±2.84^a^	6.56±0.73	6.82±0.73
H-Y stage	2.88±1.06	2.38±0.53	NA	NA
UPDRSIII score	17.37±4.91	13.50±5.09^b^	NA	NA

Measurement data are expressed as mean ± SD.

a P<0.001 compared with control groups;

b P<0.05 compared with LRRK2^+^ PD.

PD, Parkinson’s disease; LRRK, leucine-rich repeat kinase 2; MMSE, Mini-Mental Status Examination; HAMD, Hamilton Depression Scale; H-Y, Hoehn and Yahr stage scale; UPDRSIII, United Parkinson’s Disease Rating Scale; NA, not applicable.

**Table II:** Numbers of nails inserted within 30 sec by PD patients and controls in the pegboard test.

	Groups			
	
Variable	LRRK2^+^ PD	LRRK2^−^ PD	LRRK2^+^ control	LRRK2^−^ control
Single-task, PPTD	11.33±2.16^a^	11.99±1.73^a^	14.07±1.58	14.13±1.56
Single-task, PPTND	10.89±2.28^a^	11.06±1.73^a^	13.22±1.83	13.84±1.60
Single-task, PPTB	17.33±4.87^a^	17.99±3.70^a^	20.77±4.13	21.33±3.14
Dual-task, PPTD	10.39±1.82^a^	10.16±1.83^a^	13.07±1.92	13.84±1.60
Dual-task, PPTND	9.93±1.73^a^	9.37±1.42^a^	13.04±1.96	13.34±2.11
Dual-task, PPTB	16.79±3.54^a^	15.79±3.70^a^	18.77±4.13	19.53±3.85

Measurement data are expressed as mean ± SD.

a P<0.05 compared with control groups.

LRRK2, leucine-rich repeat kinase 2; PD, Parkinson’s disease. PPTD, dominant hand score; PPTND, non-dominant hand score; PPTB, both hands score.

**Table III:** Numbers of correct responses within 30 sec of PD patients and controls in the subtraction test.

	Groups			
	
Variable	LRRK2^+^ PD	LRRK2^−^ PD	LRRK2^+^ control	LRRK2^−^ control
NU	6.95±2.93	6.91±2.54	6.97±2.17	7.33±2.54
ND	3.80±1.66^a^,^b^	4.12±1.58^a^,^b^	5.66±1.59	6.48±1.04
NND	4.54±1.75^a^,^b^	5.68±1.62^a^,^b^	6.08±1.53	7.26±2.05
NB	3.47±2.31^a^,^b^	4.68±1.02^a^,^b^	8.15±2.90	8.66±2.05

Measurement data are expressed as mean ± SD.

a P<0.05 compared with control groups;

b P<0.05 compared with NU.

LRRK2, leucine-rich repeat kinase 2; PD, Parkinson’s disease. NU, single-task score; ND, subtraction test score in a dual task using the dominant hand; NND, subtraction test score in a dual task using the non-dominant hand; NB, subtraction test score in a dual task using both hands.

**Table IV:** Differences in inserted nail number and number of correct responses within 30 sec between single and dual tasks in PD patients and controls.

	Groups			
	
Variable	LRRK2^+^ PD	LRRK2^−^ PD	LRRK2^+^ control	LRRK2^−^ control
PVD	0.24±0.10	0.13±0.95	0.18±0.67	0.20±0.10
PVND	0.18±0.11	0.14±0.10	0.17±0.08	0.16±0.11
PVB	0.13±0.87	0.11±0.09	0.17±0.13	0.13±0.10
NVD	0.33±0.14^a^,^b^	0.26±0.16^a^	0.15±0.11	0.11±0.18
NVND	0.28±0.13^a^,^b^	0.22±0.16^a^	0.13±0.17	0.09±0.26
NVB	0.25±0.15^a^,^b^	0.18±0.19^a^	0.10±0.12	0.06±0.13

Measurement data are expressed as mean ± SD.

a P<0.05 compared with control groups;

b P<0.05 compared with LRRK2^−^ PD group.

LRRK2, leucine-rich repeat kinase 2; PD, Parkinson’s disease. PVD, difference in dominant hand score in the pegboard test; PVND, difference in non-dominant hand score in the pegboard test; PVB, difference in both hands score in the pegboard test; NVD, number of variation in the subtraction test score when the dominant-hand was used in the pegboard test; NVND, number of variation in the subtraction test score when the non-dominant hand was used in the pegboard test; NVB, number of variation in the subtraction test score when both hands were used in the pegboard test.

**Table V:** Analysis of correlation between UPDRSIII score and the difference in correct response number between single and dual tasks within 30 sec.

	NVD		NVND		NVB	
			
Group	R-value	P-value	R-value	P-value	R-value	P-value
LRRK2^+^ PD	0.687	0.001	0.641	0.001	0.622	0.001
LRRK2^−^ PD	0.663	0.008	0.557	0.006	0.549	0.006

LRRK2, leucine-rich repeat kinase 2; PD, Parkinson’s disease. NVD, difference in dominant hand score; NVND, difference in non-dominant hand score; NVB, difference in both hands score.
